# A lesson for cancer research: placental microarray gene analysis in preeclampsia

**DOI:** 10.18632/oncotarget.595

**Published:** 2012-08-23

**Authors:** Frank Louwen, Cornelia Muschol-Steinmetz, Joscha Reinhard, Anke Reitter, Juping Yuan

**Affiliations:** ^1^ Department of Gynecology and Obstetrics, School of Medicine, J. W. Goethe-University, Frankfurt, Germany

**Keywords:** preeclampsia, cancer cells, invasion, angiogenesis, immune tolerance

## Abstract

Tumor progression and pregnancy share many common features, such as immune tolerance and invasion. The invasion of trophoblasts in the placenta into the uterine wall is essential for fetal development, and is thus precisely regulated. Its deregulation has been implicated in preeclampsia, a leading cause for maternal and perinatal mortality and morbidity. Pathogenesis of preeclampsia remains to be defined. Microarray-based gene profiling has been widely used for identifying genes responsible for preeclampsia. In this review, we have summarized the recent data from the microarray studies with preeclamptic placentas. Despite the complex of gene signatures, suggestive of the heterogeneity of preeclampsia, these studies identified a number of differentially expressed genes associated with preeclampsia. Interestingly, most of them have been reported to be tightly involved in tumor progression. We have discussed these interesting genes and analyzed their potential molecular functions in preeclampsia, compared with their roles in malignancy development. Further investigations are warranted to explore the involvement in molecular network of each identified gene, which may provide not only novel strategies for prevention and therapy for preeclampsia but also a better understanding of cancer cells. The trophoblastic cells, with their capacity for proliferation and differentiation, apoptosis and survival, migration, angiogenesis and immune modulation by exploiting similar molecular pathways, make them a compelling model for cancer research.

## Pregnancy and malignant tumor

One of the initial processes of human pregnancy is characterized by the attachment of the blastocyst to the uterine decidua. Implantation progresses by expanding the trophoblastic cells and by their differentiation into two cell lineages, the villous- and the extravillous trophoblasts [1]. The extravillous trophoblasts, proliferative and invasive, invade into the uterine decidua to anchor the developing embryo to the uterus and to establish appropriate nutrient and oxygen supply for the fetus [1-3]. The invasion of extravillous trophoblasts into the uterine wall is of crucial importance for fetal development, and is tightly regulated in a temporal and spatial manner. Its deregulation has been implicated in a wide spectrum of abnormal pregnancies, such as preeclampsia. Strikingly, these extravillous trophoblasts display a phenotype very similar to cancer cells with their capacity for proliferation, migration, angiogenesis and immune tolerance by exploiting comparable molecular mechanisms, making them an interesting model for cancer research [1,4-6].

## Preeclampsia, a complex disorder

Preeclampsia, characterized by the new onset of hypertension and proteinuria after 20 weeks of gestation, is a consequence of diverse pathophysiological processes linked to impaired implantation, endothelial dysfunction and systemic inflammation [7-9]. It is a multisystem disorder unique to human, and affects 2-7% of nulliparous women [7]. It causes not only maternal and perinatal mortality and morbidity but also associates with long-term effects on the cardiovascular complications of mother and child. Clinically, the affected mother demonstrates increased blood pressure, proteinuria, edema, abnormal clotting, and liver and renal dysfunction, whereas fetal preeclampsia syndrome can manifest as preterm delivery, growth restriction, placental abruption and fetal distress [10]. Preeclampsia is associated with abnormal placentation, uteroplacental vascular insufficiency and altered intervillous haemodynamics, placental oxidative stress, and increased placental release of syncytiotrophoblast debris and anti-angiogenic molecules, which cause dysfunction of maternal endothelial cells and a systemic inflammatory response [8,11].

Despite intensive research, a full understanding of the pathogenesis of preeclampsia remains elusive. Several mechanisms have been implicated in the etiology of preeclampsia, including immunological abnormality, defect in vascular/ischemic modeling, deregulated inflammatory factors, lipid and metabolic disorder, failures in regulatory pathways of hormone synthesis and prostaglandin action [8]. Particularly, while immunologists consider preeclampsia as a maternal-embryonic immune maladaptation [12,13], vascularists propound that ischemia-reperfusion leads to oxidative stress and vascular disease [14,15]. Both of these aspects may be important for preeclampsia pathogenesis [16]. A two-stage model has been recently proposed in which the initiating event, poor placentation, is thought to occur early in gestation [11,17]. At this stage of preeclampsia, the most affected area of the placenta is the basal plate, where trophoblast invasion takes place. Interstitial trophoblast invasion is often shallow, and endovascular invasion does not proceed beyond the terminal portions of the spiral arterioles [18-20]. Thus, the placental development fails to meet the gestation-related fetal demands for increased blood flow. The second stage of preeclampsia is thought to be the maternal response to defected placentation, and systemic endothelial dysfunction appears to be the major picture for preeclampsia [11,17]. There are differences between early- and late onset preeclampsia regarding clinical presentation and outcome [8,16]. Histopathological examination of placenta also shows different morphological characteristics depending on the timing of disease onset [21]. Whether these differences are a reflection of differential placental gene expression is not yet clear.

The placenta, the main stage of these processes, plays a central role in preeclampsia pathogenesis [7-9,16,17]. It is generally believed that preeclampsia originates in the placenta, since preeclampsia ends with delivery and removal of placental tissue. Therefore, placenta-based investigations are of importance for understanding of disease initiation and progression. Microarray-based transcriptional profiling becomes a valuable tool for identifying disease-related genes and pathways [22]. This approach has been widely used for analysis of preeclampsia [23]. In this review, we will summarize the recent data from the gene analysis studies. Furthermore, we will focus on several interesting genes identified in these studies and analyze their potential function in preeclampsia, compared to the roles in malignant tumor cells.

## Microarray gene profiling in preeclampsia

Searching the microarray-based placental studies associated with preeclampsia in Pubmed, we have collected 18 studies from June of 2007 to June of 2012 [23-39], with 220 placenta samples from preeclampsia patients and 224 from controls. Among these studies, seven were designed with relatively comparable gestational age [23,27,28,30,31,33,34] and 9 with same mode of delivery (cesarean section) [24,25,30,31,34,36-39]. As listed in Tab. 1, the sampling sites varied and microarray plate forms were different. In addition, strategy of data analysis differed. In general, the differentially expressed genes from these studies include genes coding for multicellular structure development and differentiation, activity of immune response, angiogenic and vasculogenic responsible proteins, cell proliferation and apoptosis, inflammation related-molecules, and metabolism associated-proteins. The results from these 18 microarray-based studies are apparently diversified and even occasionally controversy concerning the genes and pathways of interest. A major part of the divergence could probably be ascribed to matching variability, such as gestational age, induction of labor, mode of delivery, maternal ethnicity, varied placental sampling sites, different types of microarray chips and platforms, distinct data filtering strategy and diverse statistical analysis.

**Table 1:** Summary of microarray studies in preeclampsia

Author	Group	Mother age	Gest. age (wks)	BMI	MOD	Sampling	Microarray	Finding	Hot genes	Pathways
Jarvenpaa 2007 Finland	2 PE + IUGR 3 ctrl	21.5 27.7	35.7 38.5	20 27.3	Cs without labor	Not described	Affymetrix Human U133 plus 2.0	4 genes up 9 genes down	Up: EPAS1, FLT1, SIGLEC10, ANG4 Down: ECGF1, JAG1, Palladin, COL18A1, TNFSF12, VEGF, ANPEP, PDGFA, SERPIN12	Angiogenesis
Centlow 2008 Sweden	10 PE 5 PE +notch 5 notch 15 ctrl	28.5±2.9 32.4±4.3 31.8±5.8 31±3.3	37.7±2.3 35.0±4.1 38.9±3.1 40.0±1.4		Cs/vag.	Villous tissue from central part of placenta, frozen on dry ice and stored at -80°C.	Operon v 2.1 human 70 mer oligo set	30 genes were altered in at least one fold between-group comparison.	Up: Hbα2, Hbγ and Hbβ in PE vs. ctrl.	Potent toxins: endothelial damage, inflammation
Toft 2008 Norway	10 PE 8 SGA 10 PE+ SGA	30.3±5.4 34.4±5.0 30.3±4.9	34.2±2.5 34.5±3.8 33.9±2.0		Cs	Tangentially dissection from the maternal side close to umbilical cord, stored at -80°C.	Affymetrix HG U133 plus 2.0	No difference between study groups.	qPCR: FLT1 and ENG are up regulated in PE+SGA group	Angiogenesis
Enquobahrie 2008 USA	18 PE 18 ctrl	32.6 30.0	35.8 38.9	27.0 25.3	10 Cs 7 Cs	Pooled of 4 samples from maternal side, stored at -80°C.	Operon Human genome array ready oligo set	1164 genes altered in PE vs. ctrl. 58 genes (56 up and 2 down regulated) had an absolute change ≥1.5.	Up: LEP, FLT1, PCDHA3, CYP11A, F2R, IL9, FCGR2B, CDO1, VGLL1, EBI3, INSL4, BCL6,INHA Down: MGC1132, NR4A2	Reproductive physiology, immune response, cytokines, cell cycle
Winn 2009 USA Denmark	12 PE 11 PTL	30.7±9.1 30.2±7.1	32.1±3.3 31.0±4.6		6 Cs/10 labored 2 Cs/11 labored	Basal plate, Snap frozen.	Affymetrix HG-U133A / HG-U133B	55 genes differentially expressed.	Up: FLT1, LEP, CRH, SIGLEC6, PAPPA2, INHA, ENG, HTRA1	Lipid metabolism, angiogenesis
Sitras 2009 Norway	16 PE severe PE 21 ctrl	30.5±5.2 30.2±4.8	34.0±3.6 39.6±1.3	25.9±4.8 24.8±5.3	11 Cs 8 Cs	Chorionic tissue, 2 cm beside umbilical cord middle layer of placenta, stored at -80°C.	Applied Biosystems Human genome survey microarray v2.0	213 genes up and 82 down in PE vs. ctrl. 36 up and 132 down in early- vs. late onset of PE.	Up: LEP, FLT1, FLT4, β-hCG, ENG, LAEVERIN, BCL6, INHA, MMP14, PAPPA2 Down: PDGFD	PE vs. ctrl: Angiogenesis, Oxidative stress, inflammation early vs. late: endothelial signaling
Founds 2009 USA (CVS)	4 PE 8 ctrl	36.5±0.6 38.1±3.1	11.4±0.7 11.3±0.6	29.9±4.2 24.5±4.0	Cs/vag.	Chorionic villous sample at 10-12 wks.	Affymetrix HG-U133 Plus 2.0 Gene Chip	5 up and 31 down in 1^st^ trimester in PE vs. ctrl.	Up: CCK, CTGA2 Down: FSTL3, MMP12, LAIR2, S100A8	Inflammation, immune regulation, cell motility
Lee 2010 Korea	13 PE severe 13 ctrl	31.85±3.93 33.08± 4.65	35.93± 0.9 38.48±0.56		Cs without labor	Central area, stored at -80°C. Pooled RNA.	Agilent Human 4X44K	121 up and 294 down in PE vs. ctrl.	Up: CXCR6, CXCL3, OSM, LEP, FLT1, VEGFA,SMOX, CYP26A, EGLN3, LDHA, CRY2L1	Angiogenesis
Hoegh 2010 Denmark	11 PE 18 ctrl				Cs/vag.	Maternal side, center of cotyledons, stored differently, Pooled RNA.	Affymetrix HG-U133A Gene Chip	12 up and 9 down in PE vs. ctrl.	Bradykinin B1 receptor, 14-3-3, INHBA, LEP	Placentation, oxidative stress, inflammation
Várkony 2011 Hungary	6 PE 6 PE+ HELLP 5 ctrl preterm 5 ctrl term	34.3 28.7 31.6 30.8	32.4 30.7 31.0 38.9	24.3 23.7 23.4 26.7	Cs	Villous tissue from central cotyledons close to umbilical cord, stored at -80°C.	Agilent 44K whole human genome oligo	181 altered in preterm vs. term, 350 in PE vs. ctrl and 554 in HELLP vs. ctrl.	Up: LEP, CGB, TREM1, LHB, SIGLEC6, PAPPA2 Down: KRT81, OPRK1	Multicellular structure, differentiation neuroactive ligand- receptor interaction
Tsai 2011 USA	23 PE 37 ctrl		33.6±3.7 37.6±1.93		Induc-tion of labor	Fetal side, 5 cm from umbilical cord, in liquid nitrogen	Illumina Human6- v2 BeadArray	128 altered in PE vs. ctrl.	ENG, PAPPA2, RDH13, INHA, LEP, FLT1, SIAE, SIGLEC6	Immune response
Chang 2011 Taiwan	13 PE 10 ctrl 7 PE super-impose	28.1±1.4 33.2±1.6 33.5±1.9	33.5±0.9 38.8±1.0 34.2±1.2		Not descri-bed	Maternal side, stored at -80°C.	Human 15K chips		Up: HSPA1B, LIMS1, PLAGL1, TRIM31, PPP2R2C Down: RNF128, ADM, ARFIP1	Development, apoptosis, cell death
Kang 2011 Korea	16 PE 17 ctrl	31.7±3.9 33.1±4.2	36.1±2.4 39.0±0.9		Cs without labor	Chorionic tissue, near umbilical cord, in liquid nitrogen.	GE healthcare Human whole genome bioarrays	132 altered, in PE vs. ctrl.	UP: FLT1, LEP ITGA5, EBI3 SIGLEC6, HTRA1	Proliferation, differentiationimmune, biosynthesis, transport of lipid or protein
Nishizawa 2011 Japan	8 PE severe 8 ctrl 8 FGR	31.0±4.7 31.5±6.5 31.4±3.7	34.4±1.8 38.1±0.8 37.3±1.0	21.7±3.7 21.4±2.3 19.9±1.9	Cs without labor	Sections of placental villi between the basal and chorionic plates, in liquid nitrogen.	Affymetrix Human Exon 1.0 ST Array	94 up and 88 down at least 1.5 fold in PE vs. ctrl. 30 up and 32 down of these in FGR.	Up: INHBA, INHA, FLRG, BCL6, LEP, UP:PAPPA2, FLT1, ENG, CGB, CRH Down: GSTA3	p53 targets, cell growth, differentiation
Mayor-Lynn 2011 USA	7 PE 7 PTL 7 ctrl	23.8(20-26) 28.3(22-35) 30(21-38)	35 (31-39) 28 (24-33) 38 (37-39)	38.2 30.8 37.5	Cs	Villous tissue.	Illumina HumanRef-12 v3 Expression BeadChip	120 altered in PE and PTL compared to ctrl.	CRH,SOCS1,MMP1, MMP9, ADAM17, ADAM30, TIMP3, STC2, CRHBP, EDN2	Inflammation, cell cycle, cell-to-cell signaling, Embryonic development
Junus 2012 Sweden	Early 8 PE 4 ctrl late: 7 PE 6 ctrl	31.5±5.0 34.0± 4.5 30.0±11.5 24±4.5	29.3±3.0 24.4±1.6 39.9±1.9 40.2±2.2		8 Cs vag. 1 Cs 2 Cs	Central part of placenta stored at -80°C.	Operon v 2.1 human 70-mer oligo set	88 up and 108 down in early- vs. late onset.	Down in early: ACVRL 1, EGFL7, ROBO4, IDO 1	Angiogenesis, cell motility, oxygen transport
Meng 2012 China	6 PE 6 ctrl	26.0±4.3 28.5±1.9	36.4±0.9 39.0±0.7		Cs without labor	Sections from maternal face of the placenta Stored at -80°C	Illumina HumanHT-12 V4 BeadChip	483 up and 456 down at least 2 fold in PE vs. ctrl.	Up: BTNL9. HMBS. ULBP1, CHRNA1, RMRP Down: INSL6, CXCL9, TMCC1, PAGE2	Cellular function and development, cell signaling, lipid metabolism
Lapaire 2012 Switzerland	9 PE severe 7 ctrl	36.8(22-43) 35.1(33-37)	34.6 (33-39) 38.6 (38-41)	26.3 (30-34) 21.1 (19-37)	Cs Cs	Villous tissue, stored at -80°C.	Affymetrix GeneChip Human 1.0 ST Arrays	896 differentially expressed.	Up: βhCG, HTRA4, CRHBP, LHB, QPCT, CD97, MMP19, ADAM2, INHBC Down: CCL3, NOX4, VCAM1, FOSB, CX3CR1	Riboflavin metabolism, leukocyte extravasation, NF-κB and chemokine signaling

Cs: Cesarean section; vag: vaginal delivery; PTL: preterm labor; PE: preeclampsia; HELLP: haemolysis, elevated liver enzymes and low platelets; SGA: small for gestational age; BMI: body mass index; MOD: mode of delivery; wks: weeks; ctrl: control; FDR: false discovery rate; IUGR: intrauterine growth restriction; FGR: fetal growth restriction; Up: upregulted; Down: downregulated.

Among these 18 microarray studies, two dealt with early and late onset of preeclampsia [29,33], and the data show that they share some genetic features yet differ in other signatures suggesting these two types might have different pathogenesis. As the alteration of genes triggering preeclampsia has been thought to take place at a very early stage of gestation, gene analysis using placental samples after delivery may include both causative factors as well as secondary responses. Of note, one of these 18 studies was based on chorionic villous samples from 10-12 gestational weeks with the interestingly results that deregulated genes are mainly involved in inflammation, immune response and cell motility [23]. The results have been underlined by another study based on chorionic villous samples yet using quantitative PCR, showing that genes linked to trophoblast invasion and utero-placental hemodynamic adaptation are altered already in the first trimester [40].

In general, the results from these studies deliver multiple pictures of gene signatures, highlighting the complicated pathophysiology and heterogeneous causes of this disease. Despite the complex and inconsistence in gene signatures, the observations from these 18 studies show nevertheless some overlapping genes and pathways associated with preeclampsia, as discussed below. As one single gene is very often involved in several molecular networks, we will discuss it only in one signaling pathway.

## I. Angiogenesis and vasculature pathway

From these 18 microarray studies in preeclampsia, the most repeatedly reported genes are fms-like tyrosine kinase 1 (FLT1) and endoglin (ENG) [25,27-29,29,30,32,34-37]. Other differentially expressed angiogenesis-related genes have also been reported, such as platelet derived growth factor A (PDGFA),[25] EGF-like-domain, multiple 7 (Egfl7) and Activin A receptor type II-like I (Acvrl I) [33], vascular endothelial growth factor (VEGF) [25,30], FLT4 and platelet derived growth factor D (PDGFD) [29]. In addition, the differentiated expression of other angiogenic genes have been also observed, including JAG1 (a signaling factor which stimulates angiogenesis in endothelial cells), ECGF1 (endothelial cell growth factor, platelet-derived) and COL18A1 (collagen, type 18, A1, a potent anti-angiogenic protein) [25]. These data underscore the recent observation that an imbalance in circulating factors with pro- and anti-angiogenic/vasculogenic functions, such as soluble vascular endothelial growth factor receptor 1 (VEGFR1, sFlt-1), placental growth factor (PlGF), and transforming growth factor co-receptor endoglin, is important in the pathogenesis of preeclampsia [41-44].

### FLT1

Members of the VEGF family are critical factors involved in placental angiogenesis and play functional roles in the adaptation mechanisms of neovasculogenesis in response to the compromised placental oxygen delivery in preeclampsia. The hypoxia-induced factor 1 (HIF-1) induces expression of angiogenesis-related genes, such as FLT1, and oxygen-regulated genes [45,46]. Two mRNAs are generated from the FLT1 gene in placenta and vascular endothelial cells, a long form for the full-length receptor FLT1 and a short form for soluble FLT1 (sFLT1), which carries only the ligand binding region [47,48]. This short form of the mRNA is generated by premature polyadenylation within intron-13 [49,50]. In the placenta, trophoblasts expressing FLT1 have much more sFLT1 than the full length FLT1 [51]. Indeed, its overexpression is repeatedly reported to be elevated in preeclampsia in these microarray-based studies, as described above, regardless of varied sampling sites, or early and late onset of preeclampsia. Enhanced expression of the FLT1 gene in preeclamptic placenta is consistent with the observation that sFlt1 is increased in the maternal circulation in women destined to develop preeclampsia [52,53]. sFlt1 binds to and antagonizes both VEGF and PlGF by preventing them interacting with their endogenous membrane receptors and induces thereby endothelial dysfunction [50,54]. In support of this idea, overexpression of sFLT1 has been shown to induce preeclampsia in rats, highlighting that sFlt1 inhibits angiogenesis and contributes to the placental insufficiency of the pathogenesis of preeclampsia. These data also imply that sFlt1 is an attractive target for treating preeclampsia, as demonstrated by a pilot study [55].

Tumor progression is invariably associated with hypoxia, a common state of cancer cells due to the lack of blood supply to the rapidly growing tumor [56]. Insufficient oxygenation has opposing effects on cancer cells: limiting tumor cell division as well as promoting tumor cell more aggressive and invasive [57]. Like trophoblasts in placenta, the response of tumor cells to oxygen deprivation is also driven by hypoxia induced factor-1 (HIF-1). HIF-1 is the key factor mediating adaptive response to hypoxia, and is degraded under normal condition by prolyl hydroxylases (PHD) and accumulated upon hypoxia [58]. Tumor cells are induced to secrete pro-angiogenic factors such as VEGF to form the new blood vessels [59], and activate an invasive program mediated by the c-Met/HGF (hepatocyte growth factor) pathway [60]. Therefore, like in normal pregnancy, the VEGF-VEGFR system plays critical roles in tumor progression, making it an interesting target for cancer therapy. Blocking the VEGF-VEGFR system, like the function of sFlt1 in preeclampsia, has been regarded as a powerful strategy for cancer therapy. In fact, the anti-VEGF neutralizing antibody bevacizumab and multi-tyrosine kinase inhibitors such as sorafenib and sunitinib have been developed and are widely used in the treatment of cancer [61,62]. In most cases, these agents improve progression-free survival and overall survival. However, frequent side effects of these anti-VEGF-VEGFR agents are hypertension and proteinuria, indicating a close relationship between deregulated VEGF and hypertension/proteinuria, characteristic of preeclampsia [63]. The data also suggest that desired agents should target selectively tumor-related angiogenesis [64], not the systemic VEGF function.

### ENDOGLIN (ENG)

In addition to FLT1, the ENG gene is also increased in the preeclamptic placenta, reported by several groups [28,29,32,34,35,37]. ENG codes for endoglin, which is a co-receptor for transforming growth factor β1 and β3 (TGF-β1 and TGF-β3) on cell membranes of endothelium and syncytiotrophoblast cells. In endothelial cells, TGF-β signals activate the Smad-dependent as well as the Smad-independent signaling pathway, to regulate fundamental cellular processes, including proliferation, differentiation, migration, apoptosis, adhesion, cytoskeletal organization and extracellular matrix remodeling [65,66]. Endoglin is highly increased in the maternal serum of preeclamptic women and may cooperatively act with sFlt1 to induce severe preeclampsia [67]. Its level falls after delivery and correlates with disease severity [67]. Endoglin decreases endothelial nitric oxide signaling by inhibiting TGF-β1, leading to endothelial dysfunction [67,68]. Moreover, endoglin activates eNOS [68,69] as well as interacts and modulates Activin receptor-like kinase-1 and -5 signaling [69], resulting in the potentiation of Smad1 and Smad2 and inhibition of Smad3 [69,70], thereby disrupting homeostasis and causing the development of preeclampsia [69]. In addition, endoglin decreases arterial diameter of rat renal micro-vessels by modulating TGF-β1 and TGF-β3 mediated vasodilation [67]. Soluble endoglin cooperates with sFlt1 to induce endothelial dysfunction in human umbilical vein endothelial cells (HUVECs), and simultaneous administration of both causes a severe preeclampsia-like illness in rats [41,52,67]. Taken together, the data suggest that endoglin plays an important role in the pathogenesis of preeclampsia.

Interestingly, ENG expression strongly results in active vascular endothelial cells in tumor [71]. Endoglin in the blood vessel endothelium is involved in the control of cell proliferation, migration and capillary tube formation, and plays a pro-angiogenic role in tumor development [71,72]. Furthermore, endoglin suppresses cancer metastasis, which is associated with decreased expression in several Smad1-responsive genes [73]. It appears that tumor cells and trophoblastic cells exploit the same angiogenesis mechanisms involved in tumor progression and in placental development in pregnancy. Endoglin has been also suggested as an appropriate marker for tumor-related angiogenesis and neovascularization and numerous studies demonstrate the potential of endoglin in tumor diagnosis, prognosis and therapy [71]. Compared to Flt1, endoglin is possibly a more specific target for cancer therapy, since its expression is more tumor-related. Thus, targeting endoglin has been attracting high attention as a promise therapeutic strategy in human malignancies. Among therapeutic agents, monoclonal antibodies have shown anti-tumor efficacy. Several studies demonstrate a long-lasting suppression of tumor growth and metastasis in immune deficient mice by administrating antibodies [74,75], possibly by inhibiting tumor-associated angiogenesis, and/or by destructing tumor-associated vasculature. Recently, it has been shown that TRC105, a chimeric IgG1 monoclonal antibody binding to endoglin, inhibits tumor angiogenesis and appears to have a safety profile in a clinical phase I trial [76]. Importantly, classic toxicities associated with VEGF inhibition, including hypertension, proteinuria and thrombosis were not prominent [76], suggesting that endoglin is a selected target for debating tumor-associated angiogenesis.

### PlGF

Interestingly, no distinct differential expression of the placental growth factor (PlGF) was reported from these 18 studies. PlGF is a pro-angiogenic protein and member of the VEGF family produced by villous syncytiotrophoblasts in the placenta [77]. Previous studies have demonstrated that levels of circulating PlGF in the serum of patients with preeclampsia are significantly decreased compared with normotensive controls [41,78]. This discrepancy is possibly ascribed to the fact that PlGF decreases in the maternal circulation already several weeks before the onset of preeclampsia [79], or alternatively, the down-regulation of PlGF happens only in a minor population of trophoblasts, which is difficult to be reflected by global gene analysis with whole cell populations of placenta.

### II. Immune and defense response

Active avoidance of tumor cells from elimination by immune cells is one of cancer hallmarks [80] and restored antigenicity and immunogenicity of tumor cells by agents, such as metformin [81], may represent a novel strategy for cancer prevention and treatment. Like in tumor progression, immune tolerance is of critical importance for placental development and deregulated immune response is associated with pathogenesis of preeclampsia. Based on the results from these 18 studies, one of the most striking genes is sialic acid binding Ig-like lectin 6 (SIGLEC6), which is generally elevated [27-29,31,32,36]. Other differentially expressed genes involved in immune and defense response include sialic acid acetyl esterase (SIAE) and ST6 beta-galactosamide alpha-2,6-sialyltranferase 1 (ST6GAL1) [32], and Epstein-Barr virus induced gene 3 (EBI3) [27,36,37,39]. Interestingly, B-cell CLL/lymphoma 6 (BCL6) is shown to be increased [27,37]. In addition, 31 genes involved in the CXC chemokine receptor 4 (CXCR4) signaling were differentially expressed in preeclamptic placentas, compared to normal controls [32]. Finally, other immune and defense response related-genes are also reported to be deregulated, such as FCGR1A, FCGR2B, IL9, NR4A2, PROCR and IFIT4 [27]. Collectively, the genes associated with immune response are obviously altered in preeclampsia.

### SIGLEC6

Siglecs, sialic acid binding immunoglobulin-like lectins, are a family of cell surface receptor proteins which recognize sialylated glycans and transmit signals to immune cells, regulate cellular activation in the immune system [82,83]. They are characterized by an N-terminal V-set immunoglobulin domain that mediates sialic acid binding followed by varying numbers of C2-set immunoglobulin domains [84]. Siglec6 was originally identified both as a leptin-binding protein (obesity binding protein-1 or OB-BP1) and as a placental protein [85,86]. Further analysis demonstrates that high expression of Siglec6 is found to be strictly only on cyto- and syncytiotrophoblasts in placenta and on B cells in human [86]. Its cytosolic part contains the immunoreceptor tyrosine-based inhibitory motif (ITIM) and the ITIM-like domains. Receptors with ITIM function as inhibitory receptors via recruitment of cytoplasmic phosphatase with Src homology 2 (SH2) domain [87]. The molecular function of Siglec6 in placenta is not yet understood. It has been implicated in being associated with regulating cellular activation within the immune system [84], and could play a role in term labor [88]. Recently, an interesting study shows that Siglec6 interacts with glycodelin-A (GdA) to suppress trophoblast invasiveness by inhibiting extracellular signal-regulated kinase (ERK)/c-Jun signaling pathway [89].

Interestingly, in spite of varied sampling sites or early/late onset of preeclampsia, increased gene expression of SIGLEC6 is repeatedly observed in microarray gene profiling studies in preeclamptic placenta [27-29,31,32,36], implying deregulated SIGLEC6 may be tightly associated with pathogenesis of preeclampsia. Given that Siglec6 is to be found only in human placenta and preeclampsia is regarded as a uniquely human disease [90], the molecular roles of Siglec6 in villous cytotrophoblasts and syncytiotrophoblasts, and in extravillous cytotrophoblasts, in association with leptin, warrant further and deep investigations, in context of invasion process of cytotrophoblasts and immune defense response in decidua. Moreover, it is intriguingly to address whether the deregulated GdA/Siglec-6/(ERK)/c-Jun network could be responsible for the shallow invasion characteristic in preeclampsia placenta [89]. Importantly, the gene and protein level of Siglec6 is specifically increased in primary pulmonary mucosa-associated lymphoid tissue (MALT) [91]. Whether it has a role in the pathogenesis of the MALT remains to be clarified.

### EBI3

EBI3, involved in immune/defense response, is an interesting gene differentially expressed in preeclamptic placentas [27,36,37,39]. Trophoblasts are thought to play a key role in maternal tolerance to the semiallogeneic fetus, in part through cytokine production and natural killer cell interaction. EBI3 encodes a soluble hematopoietin receptor related to the p40 subunit of interleukin-12 (IL-12) [92]. It is highly expressed in placental cells in pregnancy, including syncytiotrophoblasts and extravillous trophoblasts [93]. In addition, EBI3 levels are strongly up-regulated in circulation of pregnant women and gradually increased with gestational age. These data, together with the finding that EBI3 peptide is presented by HLA-G, suggest that EBI3 is a critical immune modulator in the fetal-maternal relationship. Moreover, EBI3 has been also shown to associate with p28 to form IL-27, a heterodimeric cytokine with important immune regulatory functions [94]. It has been reported that syncytiotrophoblast cells as well as extravillous trophoblast cells invading the decidua are found to express the both peptides of IL-27, namely EBI3 and p28, suggesting that IL-27 may be linked to the cytokine network regulating local immune responses in pregnancy [95]. As EBI3 expression in extravillous trophoblast cells is to be tightly regulated, it is important to investigate the role of increased EBI3 in preeclampsia.

EBI3 has roles not only in maintenance of pregnancy or immune tolerance of the human maternal body toward the fetus, but also in Hodgkin's lymphoma and adult T-cell lymphoma/leukemia [96,97]. Moreover, EBI3 is also found to have a crucial role in human lung cancer development and is implied to be responsible for growth and malignancy of lung tumors [98]. However, the detailed molecular role of EBI3 in oncogenesis is not understood. It is tempting to speculate that, combined with p28 to form IL-27, expression of EBI3 is maybe linked to immune response in cancer cells. It is thus of importance to study the association of EBI3 expression with other human malignancies and its molecular roles in tumor cell development.

## III. Motility and invasion

Superficial invasion of extravillous trophoblasts and impaired spiral artery remodeling are hallmarks in preeclampsia. Invasion of extravillous trophoblastic cells into maternal uterine tissues is essential for successful placental development and progression of pregnancy. Whereas endovascular trophoblasts migrate into the maternal spiral artery for remodeling, interstitial trophoblasts invade the decidual stroma and communicate with its diverse cell types, such as stromal cells, macrophages and uterine natural killer cells, for a supportive microenvironment [99]. In contrast to malignant cells, trophoblast invasion and spiral artery remodeling are tightly controlled. Many pathways observed in cancer cells are used by invasive extravillous trophoblasts, like PI3K/AKT/mTOR, STAT/JAK, Notch signaling and integrin/FAK/Rho pathways [99]. A great body of studies demonstrates that these pathways are altered in preeclampsia. Among these 18 studies, in combination with previous data from microarray gene profiling, several studies pointed out that the factors responsible for motility and invasion of trophoblasts were altered, in particular, matrix metalloproteinases (MMPs) and tissue inhibitor of metalloproteinases (TIMPs), such as MMP1, -9 and S100A9 [39], MMP14 [28], MMP12, S100A8 [23], MMP10, -13, -15, TIMP2 and -3 [100]. The MMPs are a family of enzymes involved in degradation of extracellular matrix and have critical roles in trophoblast invasion. However, the results of one study could not be repeatedly reported by another, which could be resulted from varied placental sampling sites and gestational age. On the other hand, MMPs have long been thought to be essential for basement-membrane penetration during metastasis of cancer cells, and are especially associated with highly aggressive late-stage tumors with poor clinical outcome [101]. To understand the invasion working mechanism of trophoblasts in placenta, which is altered in preeclampsia, is doubtlessly useful for providing new targets for cancer intervention.

## VI. Apoptosis, cell survival and differentiation-related genes

Apoptosis, programmed cell death, is critical for normal placental development, including placental invasion, cytotrophoblast fusion, and syncytiotrophoblast function as well as trophoblast-mediated spiral artery remodeling in placenta. Increased apoptosis is associated with preeclampsia, possibly trigged by hypoxia and reactive oxygen [102]. It has been reported that the activity of caspase-3, the downstream effector of intrinsic and extrinsic apoptosis pathways, is increased [102], whereas BCL-2, an important inhibitor of apoptosis, is decreased in syncytiotrophoblast cells [103] and extravillous trophoblasts [104]. Moreover, Fas and FasL are altered in villous trophoblast cells in preeclampsia [105]. In addition, Smac (second mitochondria-derived activator of caspase) was significantly elevated, evidenced by the staining in syncytiotrophoblasts, cytotrophoblasts and endothelial cells in preeclampsia [106]. By contrast, XIAP (the X-linked inhibitor of apoptosis protein) and survivin, two critical inhibitors of apoptosis, were not altered in preeclampsia [106].

Intriguingly, only one among the 18 microarray studies observed that apoptosis-related genes, BAX, FASLG and p53AIP, were up-regulated in preeclampsia [37], in line with a previous study that genes coding for caspase-10, death-receptor 3 were altered [107]. Yet, other gene analysis studies could not make the same or similar observations. The data imply that the alteration of apoptosis gene expression could take place at very early stage of preeclampsia, or these genes are differentially expressed only in a small population of placental cells, which is not evident with whole RNAs from highly heterogeneous cell types of placenta. This issue could be addressed by using laser-based technology to select homologous single cells for gene microarray analysis. Alternatively, deregulated apoptosis-associated proteins, observed by immunohistochemistry as described above, come not from altered gene expression, rather from varied post-transcriptional modification, like some microRNA, not affecting the gene level but interfering with translation; or post-translational modifications, such as phosphorylation and ubiquitination, which impact protein stability, localization, interaction partners and molecular function. In addition, aponecrosis, incomplete execution of apoptosis followed by degeneration via necrosis, may get more attention in delineating the pathogenesis of preeclampsia [108], as it allows syncytial knots to keep the ability of inducing inflammatory response of the mother, characteristic for preeclampsia.

### BCL6

BCL6 is the master regulator of the germinal center reaction and a key oncogene in B cell lymphomagenesis [109]. It is a transcriptional repressor which recruits the repression machinery directly or through several co-repressors into the regulatory regions of its targets. The biological roles of BCL6 in normal B cell development and lymphoma oncogenesis have been intensively studied by the identification of the full set of genes that are targets of its transcriptional regulatory function [109]. This set of BCL6 targets points to a number of cellular functions which are likely to be controlled by BCL6 during germinal center development, including survival and DNA-damage response genes, like TP53, ATR (ataxia telangiectasia and Rad 3 related) and CHEK1 (checkpoint kinase 1), and cell cycle arrest gene CDKN1A/p21 [109-113]. BCL6 prevents thereby premature activation and differentiation of germinal center B cells and provides an environment tolerant of the DNA breaks associated with immunoglobulin gene remodeling mechanisms involved in the production of high-affinity antibodies of different isotypes [114]. Moreover, BCL6 function appeared to be relevant on a number of not previously considered cellular pathways by modulating signaling through Toll-like receptors and Wnt signaling [109,115]. Of importance, deregulated BCL6 expression is associated with lymphoma genesis, at least in part by allowing the occurrence of genetic aberrations in an environment unresponsive to DNA damage checkpoints and by interfering with the differentiation processes [114].

Although the BCL6 gene is increased in preeclamptic placentas [27,37], its biological function remains to be elucidated. It is tempting to speculate that increased BCL6 in preeclampsia may be associated with deregulated DNA-damage response, cell cycle arrest, cell survival, cell differentiation and immune response in trophoblast cells. It is of importance to find out its protein levels in different gestational stages, subcellular localization, function and its involvement in molecular networks, which could provide a better understanding for preeclampsia pathogenesis. In particular, it is intriguing to explore the DNA-damage response and repair signaling in cytotrophoblast in the presence of hypoxia and oxidative stress, in context of highly expressed BCL6 in preeclamptic placenta.

### INHIBIN A (INHA)

Another important gene reported by several studies is INHA [27,28,32,36,37,116]. Inhibin A is a growth and differentiation factor belonging to the TGF-β superfamily. It is a dimeric, disulfide-linked glycoprotein hormone, consisting of an α-subunit and one of several β-subunits, including inhibin A (a-βA) and inhibin B (a-βB) [117]. Previous studies have reported that inhibin A level was significantly elevated in the maternal serum of preeclampsia patients [118,119]. It is mainly derived from placental trophoblasts during pregnancy [120], as maternal concentrations of inhibin A are quickly decreased to a low level after the removal of the placenta [121]. It has been considered that increasing inhibin A may be induced by syncytiotrophoblastic inflammatory cytokines or placental oxidative stress in preeclampsia [122]. The increase in inhibin A seems more likely to be a placental compensation mechanism in preeclampsia to adjust the placental function [123].

### LEPTIN (LEP)

Finally, the most strikingly gene altered in preeclampsia is LEP, which is up-regulated reported by multiple microarray studies [27-32,36,37], supporting the previous results [124-126]. In accordance with this finding, leptin is increased in circulation of preeclampsia patients [127-129]. It is produced by adipocytes both in adult and fetus, and is highly expressed in placenta, which is not affected by maternal body mass index [29]. Functionally, leptin activates its receptor signaling pathways in trophoblast cells and exerts an anti-apoptotic and proliferative effect on human placenta [130]. Moreover, leptin stimulates protein synthesis by activating the translational machinery [130]. Interestingly, it has been recently shown that leptin may promote the ability of endothelial progenitor cells to participate in vascular remodeling [131]. Thus, increased production of leptin, in combination with human chorionic gonadotropin (hCG) and other angiogenic factors secreted by the poorly perfused preeclamptic placenta, may be a compensatory mechanism against endothelial dysfunction observed in preeclampsia. However, a clear picture of how an increased leptin is linked to the pathogenesis of preeclampsia remains to be elucidated.

On the other hand, leptin is also associated with malignancy development, in particular, in breast cancer. Interestingly, it is well recognized that obesity is associated with increased risk of more aggressive breast cancer as well as reduced survival of the cancer patient [132,133]. Obesity is associated with increased inflammation, angiogenesis, and alterations in serum levels of potential growth regulators [134,135]. Both the adipocytes as well as the non-adipocyte fraction of the adipose tissue synthesize and secrete several adipokines including leptin, tumour necrosis factor (TNF)-α, interleukin-6 (IL-6) and hepatocyte growth factors (HGF), which are also well known to be involved in the pathogenesis of preeclampsia. Leptin exerts its metabolic effects as well as biological activities, such as cell proliferation, apoptosis and survival on breast cancer cells [136,137].

## SUMMARY

**Figure 1:**
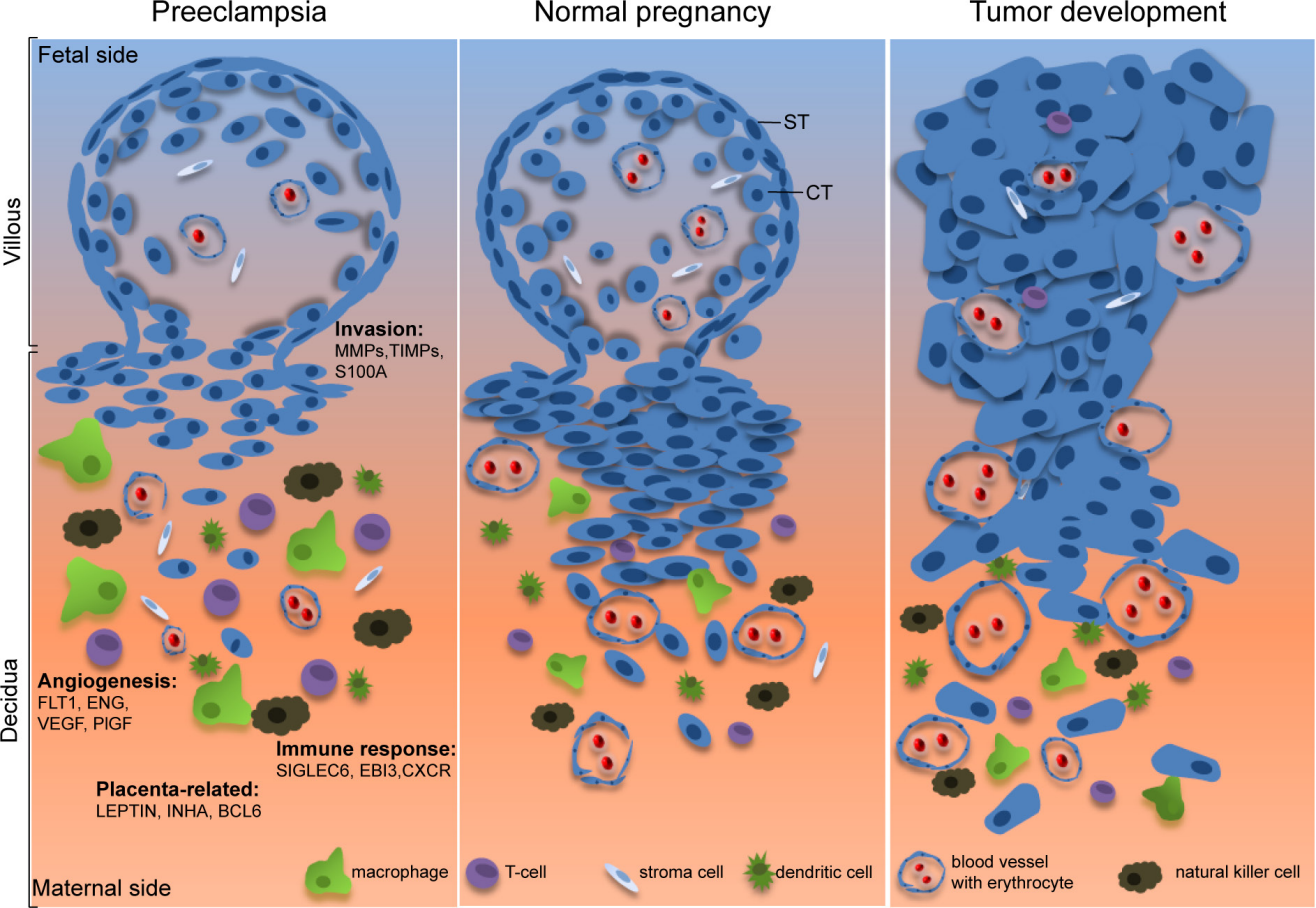
Illustrative scheme of the common features shared by normal pregnancy and tumor development While extravillous trophoblasts in the placenta are capable of migrating into the uterine decidua to establish appropriate nutrient and oxygen supply for the fetus development, malignant tumor cells have the capability to successfully invade their neighboring tissues to set up the friendly microenvironment for further progression. Moreover, both of them have the competence to establish effectively angiogenesis and to escape successfully the host immune defense system. In preeclampsia, however, trophoblasts fail to fulfill these tasks by showing defective invasion, altered angiogenesis and violent immune response. The interesting genes identified from the 18 microarray-based placental gene profiling are listed and described in the text. ST, syncytiotrophoblast; CT, cytotrophoblast. The figure design is inspired by Holtan et al, [4].

Taken together, the data from these 18 microarray-based studies display multiple pictures of gene profiles, highlighting the heterogeneous pathophysiology of preeclampsia. Despite the complex of gene signatures, these studies demonstrate that a number of genes associated with angiogenesis and immune response are differentially expressed in preeclamptic placenta, compared to normotensive placenta. Importantly, most of these identified genes, such as FLT1, EBI3, LEP and BCL6, are highly involved in angiogenesis and immune modulation in malignant tumor progression. Intriguingly, genes involved in apoptosis, proliferation and inflammation are less reported among these microarray studies, not supportive of the results from immunohistochemistry or Western blots. Further studies are warranted to corroborate the involvement of specific genes identified in these studies by using more strict design with comparable parameters, like gestational age, probe sampling and data analysis strategy. Molecular understanding of each interesting gene in signaling pathways, such as TGF-β, Wnt, Notch, STAT and VEGF related-pathways, may provide novel tools for designing new prevention and therapy for preeclampsia. Moreover, as illustrated in Fig. 1, many molecular mechanisms in trophoblastic cells, linked to migration and invasion, angiogenesis, immune tolerance, proliferation and differentiation, apoptosis and survival, are also exploited by malignant cells to set up supportive environment, to evade apoptosis and to escape the host immune response [4]. The molecular mechanisms which surrender trophoblasts immobile and trigger baneful immune response in preeclamptic placenta will shed new light on cancer research.
